# Use of Pentoxifylline to Improve Seminal Parameters in Dogs

**DOI:** 10.1002/vms3.70925

**Published:** 2026-04-11

**Authors:** Vincenzo Cicirelli, Matteo Burgio, Lorenza Frattina, Francesco Caprio, Domenico Robbe, Federica Ariu, Annalisa Rizzo

**Affiliations:** ^1^ Department of Veterinary Medicine University of Bari Valenzano Italy; ^2^ Department of Veterinary Medicine University of Teramo, Piano d'Accio Teramo Italy; ^3^ Department of Veterinary Medicine University of Sassari Sassari Italy

**Keywords:** canine semen, sperm motility, sperm parameters, sperm viability, subfertility

## Abstract

**Objective:**

The study aimed to evaluate the effect of a one‐shot oral administration of pentoxifylline on semen parameters in normospermic and oligospermic dogs.

**Methods:**

Twenty‐nine dogs were categorized as normospermic or oligospermic based on baseline sperm concentration. After 10 days from the first semen evaluation, each dog received 10 mg/kg of pentoxifylline orally, given 40 min prior to ejaculation. Semen was evaluated using a Computer‐Assisted Sperm Analysis (CASA) system and Eosin–Nigrosin staining for vitality.

**Results:**

Compared with baseline, pentoxifylline treatment resulted in a statistically significant increase in sperm concentration and progressive motility (*p* < 0.01), most notably in oligospermic dogs.

**Conclusion and Clinical Significance:**

These findings highlight the beneficial effects of pentoxifylline and confirm its potential as an adjunct therapy for improving semen quality in subfertile male dogs.

## Graphical abstract

A single oral dose of pentoxifylline (10 mg/kg) administered 40 min before ejaculation significantly improved sperm concentration and progressive motility in dogs. The effect was most pronounced in oligospermic subjects, supporting pentoxifylline as a rapid, well‐tolerated adjunct therapy for canine subfertility

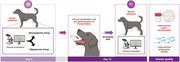

## 1 Introduction

The reproductive capacity of male dogs depends on the integrity and function of internal genital organs (testes, epididymis, ductus deferens, prostate) and external genitalia (scrotum, penis, prepuce). Infertility in dogs can result from congenital, infectious, inflammatory, neoplastic, endocrine or behavioural causes (Cicirelli et al. 2022). Common abnormalities include azoospermia (the complete absence of spermatozoa in the ejaculate, confirmed by at least two separate semen analyses performed) and oligospermia (a reduced concentration of spermatozoa in the ejaculate, below the reference thresholds) (Malmo 2003; Cicirelli et al. 2021).

Azoospermia may be pre‐testicular (e.g., fever, hernia, endocrine disorders), testicular (e.g., bilateral cryptorchidism, trauma, germ cell aplasia) or post‐testicular (e.g., epididymal aplasia, spermatocele, granulomas) (Romagnoli 2006). Differentiation from incomplete ejaculation can be achieved by evaluating seminal carnitine and alkaline phosphatase (< 2000 U/L) (Memon 2007). A hereditary component has been proposed in some breeds, such as Labrador Retrievers and Scottish Terriers (Romagnoli 2006). Oligospermia (< 100 million sperm/mL) may result from infections, inflammation, BPH, obesity, trauma, ductal obstruction, toxins, aging or endocrine disorders (Cicirelli et al. 2021; de Souza et al. 2015; Domoslawska et al. 2019; Domosławska and Zdunczyk 2020; Gradil et al. 2006); It is important to note that total sperm count per ejaculate is also considered, with values below approximately 200 million spermatozoa per ejaculate often associated with subfertility, though breed and size variations exist (Burgio et al. 2024; Hallberg et al. 2024; Tesi et al. 2018). Chronic orchitis and epididymitis may progress to azoospermia despite antibiotic treatment (Romagnoli 2006).

Several therapeutic approaches have been developed to address these reproductive disorders, with particular emphasis on strategies aimed at enhancing sperm function and fertility potential (Isidori et al. 2006).

The role of cyclic adenosine monophosphate (cAMP) as a central second messenger in sperm physiology has been extensively demonstrated: increased intracellular levels promote not only motility and kinematic performance but also facilitate agonist‐induced acrosome reaction and fertilization competence, particularly in subfertile or cryopreserved samples (Esteves et al. 2007).

Pentoxifylline, a methylxanthine derivative, enhances sperm motility and concentration by inhibiting phosphodiesterases, leading to an increase in intracellular cAMP, reduction of reactive oxygen species (ROS) and enhanced ATP production (M. Nazari et al. 2022). It also improves acrosome responsiveness, zona pellucida binding and promotes tyrosine phosphorylation of the sperm tail, a biochemical modification associated with sperm capacitation and progressive motility (Forte et al. 2025; Naz and Rajesh 2004).

Moreover, pentoxifylline has been shown to possess ROS‐scavenging properties, mitigating oxidative stress that otherwise leads to lipid peroxidation, DNA fragmentation and impaired fertilization potential (Zhang et al. 2005).

In cryopreservation contexts, pre‐freeze exposure to pentoxifylline has been associated with reduced acrosome loss during thawing and improved acrosomal reactivity post‐thaw, further supporting its use in assisted reproduction settings (Mirshokraei et al. 2011). Clinical and experimental studies, in human (Ohn et al. 2002), in ovine (Hassanpour et al. 2010) and in dog (Mirshokraei et al. 2011), have confirmed its positive effects on motility and fertilization rates, particularly in cases of asthenozoospermia.

Following oral administration in dogs and humans, pentoxifylline is rapidly absorbed, reaching peak plasma concentrations within 0.29–0.41 h, but displays low oral bioavailability (20%–30%) and is predominantly eliminated via the kidneys (Smith et al. 1986). Although its in vitro benefits on canine semen motility and longevity have been established (Koutsarova et al. 1997), there is a lack of in vivo studies addressing the efficacy of oral pentoxifylline in improving semen quality in dogs.

Based on this background, the present study evaluates the in vivo impact of a single oral dose of pentoxifylline (10 mg/kg), administered 40 min prior to ejaculation, on multiple semen quality parameters in normospermic and oligospermic dogs.

## 2 Materials and Methods

### 2.1 Animals and Experimental Design

Twenty‐nine clinically healthy, client‐owned male dogs (8–48 kg; 2–7 years) of various breeds (Table 1) were recruited and prior to inclusion, each subject underwent:

‐ Collection of anamnestic data, with a focus on reproductive history;
‐ A preliminary clinical examination to assess their general health status with baseline haemato‐biochemical profiling;
‐ Reproductive examination with scrotal and prostatic ultrasonography to rule out any potential anatomical abnormalities.

**TABLE 1:** Description of dog breeds included in the study.

N°	Breed
17	Mixed breed
2	English Pointer
1	Deutsch Kurzhaar
3	German Shepherd Dog
1	Rhodesian Ridgeback
1	Labrador Retriever
1	Dachshund
1	Akita Inu
1	Deutscher Boxer
1	Beagle
1	Jack Russel Terrier

All animals with systemic or reproductive system pathologies were not enrolled in the study. All dogs were tested for manual semen collection without exhibiting signs of stress.

An initial semen collection (T0) was performed in a dedicated collection room maintained at 22°C–24°C. Ejaculates were immediately transferred to pre‐warmed tubes at 37°C. Based on sperm concentration measured at T0, dogs were classified as:

‐ Normospermic: > 100 × 10^6^ spermatozoa/mL
‐ Oligospermic: < 100 × 10^6^ spermatozoa/mL

After a 10‐day period, each dog received 10 mg/kg of pentoxifylline orally (TRENTAL 400 mg, Sanofi Aventis, Paris France) (Marsella and Nicklin 2000) to ensure accurate dosing and rapid absorption. Food was withheld for 4 h before and after administration to avoid interference with drug uptake.

**FIGURE 1:**
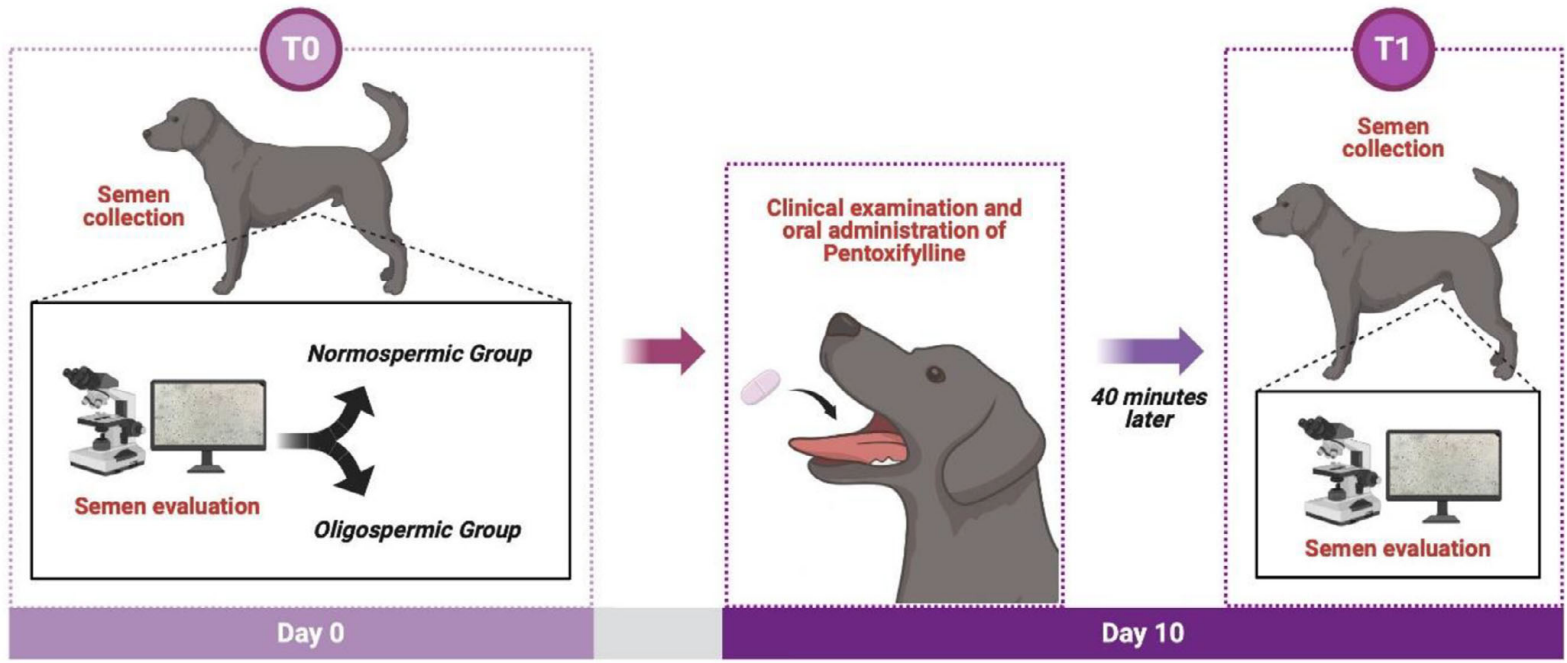
Experimental design.

A second semen collection (T1) was carried out 40 min post‐treatment using the same protocol and environmental conditions (Figure 1). All procedures were performed by the same operator to reduce inter‐operator variability.

### 2.2 Semen Collection and Analysis

The semen samples were obtained through manual collection, using a bitch in oestrus as a stimulatory aid. Semen was collected into sterile graduated tubes and separated into pre‐spermatic, spermatic and post‐spermatic fractions. Only the sperm‐rich fraction of the ejaculate was considered for evaluation, as it represents the portion containing the highest concentration of motile spermatozoa, while pre‐spermatic and post‐spermatic fractions were excluded from the analyses.

The following macroscopic parameters were recorded at both T0 and T1. A fraction of each ejaculate was diluted 1:20 in pre‐warmed Biggers–Whitten–Whittingham (BWW) medium and analysed with Computer‐Assisted Sperm Analysis (CASA; IVOS II, Hamilton Thorne, USA). The CASA software (version IVOS 12.3) used in this study for semen analysis was configured according to canine‐specific sperm parameters, as shown in Table 2.

**TABLE 2:** IVOS 12.3: Specific settings for canine sperm parameters.

Parameter	Cut‐off value
Frames per second (Fps)	30
Frequency	60 Hz
Temperature	37°C
Minimum contrast	75
Minimum cell size	4 pixels
Progressive cell threshold	100 µm/s; 75%
Lower threshold for average path velocity (VAP)	9 µm/s
Lower threshold for straight‐line velocity (VSL)	20 µm/s

The semen was placed on a Leja slide (four chambers) with a depth of 20 µm (Leja Products B.V., Nieuw Vennep, the Netherlands) and positioned in the dedicated microscope chamber, allowing a few minutes before analysis. The CASA system scanned six non‐consecutive, randomly selected microscopic fields. The evaluated parameters included sperm concentration expressed in millions per millilitre and percentage, total sperm motility in millions per millilitre and percentage, progressive sperm motility in millions per millilitre and percentage. Morphology analysis and velocity distribution (rapid, medium, slow and static spermatozoa) were also evaluated.

Sperm vitality was assessed using the Eosin–Nigrosin staining protocol as described in the World Health Organization's laboratory manual for semen analysis (WHO 1999).

**FIGURE 2:**
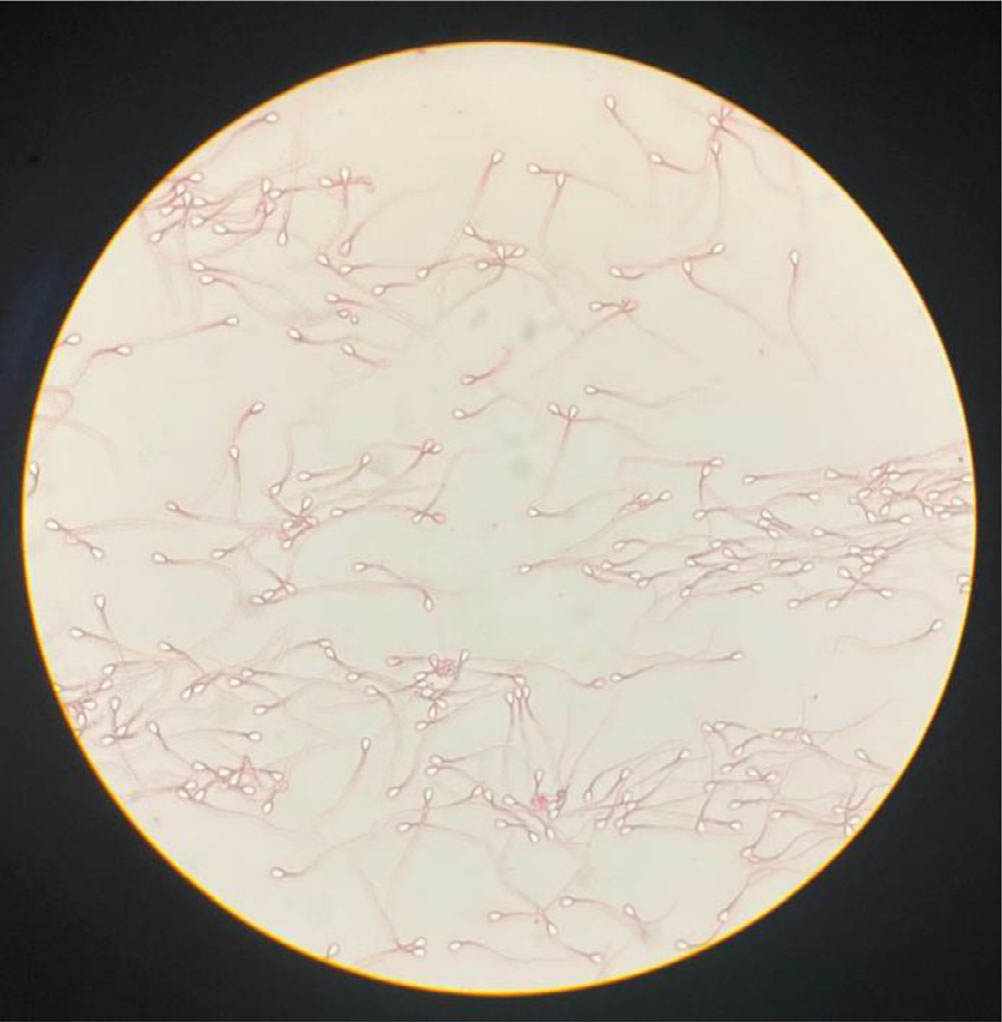
Eosin–Nigrosin staining to assess sperm vitality.

Following canine semen collection, a small aliquot of the sample was mixed with an equal volume of the staining solution, prepared by combining 0.5% Eosin Y and 10% Nigrosin in a 1:10 ratio. The mixture was gently blended and rapidly smeared onto a clean slide using the classical two‐slide technique, producing two thin smears that were air‐dried for 1–2 min. Once dried, the slides were examined under light microscopy at 400× magnification, with 100 spermatozoa evaluated per slide, consistent with previous studies (Alonge et al. 2019). Vitality as determined based on staining uptake: live spermatozoa remained unstained or lightly coloured, whereas dead spermatozoa exhibited pink or red staining due to compromised cell membranes permitting eosin penetration (Watts 2019; Figure 2).

### 2.3 Statistical Analysis

All data were collected in an excel spread sheet and statistical analysis were performed using Python software (version 3.11.0). All data were expressed as mean ± standard deviation (SD). The normality of the distribution for each parameter was assessed using the Shapiro–Wilk test. Depending on the distribution of the results, Bartlett's test (for normally distributed data) or Levene's test (for non‐normally distributed data) was applied to verify the homogeneity of variances. Based on both distribution and variance homogeneity, either the paired *t*‐test or the Wilcoxon test was used to compare time points (T0 vs. T1) within each group. While comparisons between groups (at the same time points, T0 and T1) were performed using the Student's *t*‐test (for normally distributed and homogeneous variance) or the Mann–Whitney test (for non‐normally distributed data). A *p*‐value below than 0.05 was considered statistically significant.

## 3 Results

**FIGURE 3:**
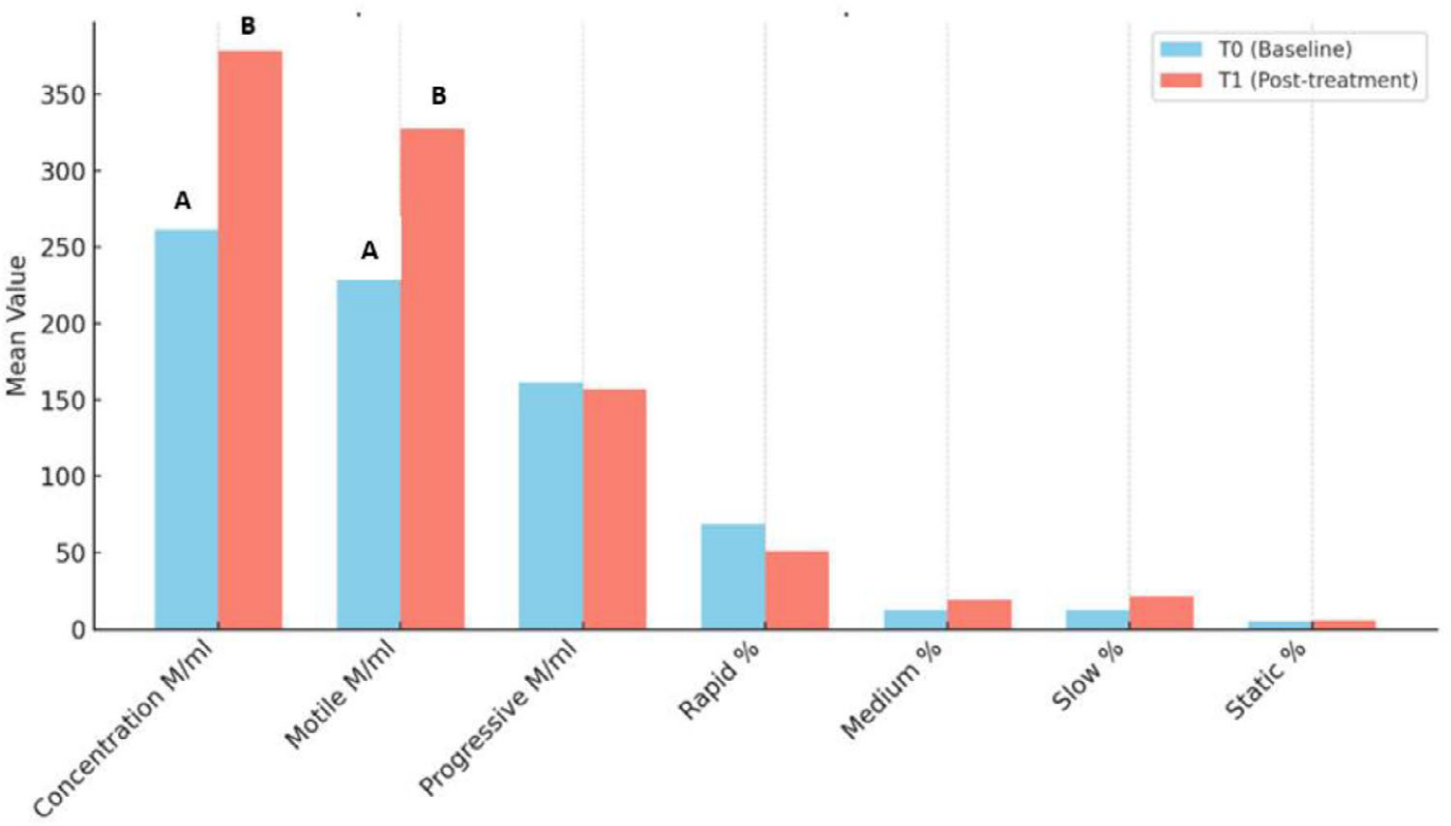
Comparative bar chart of seminal parameters at T0 and T1. Mean values of sperm concentration (M/mL), motile and progressively motile spermatozoa (M/mL), and percentages of rapid, medium‐speed, slow and static spermatozoa are compared between baseline. (A) and (B): *p* < 0.05.

**FIGURE 4:**
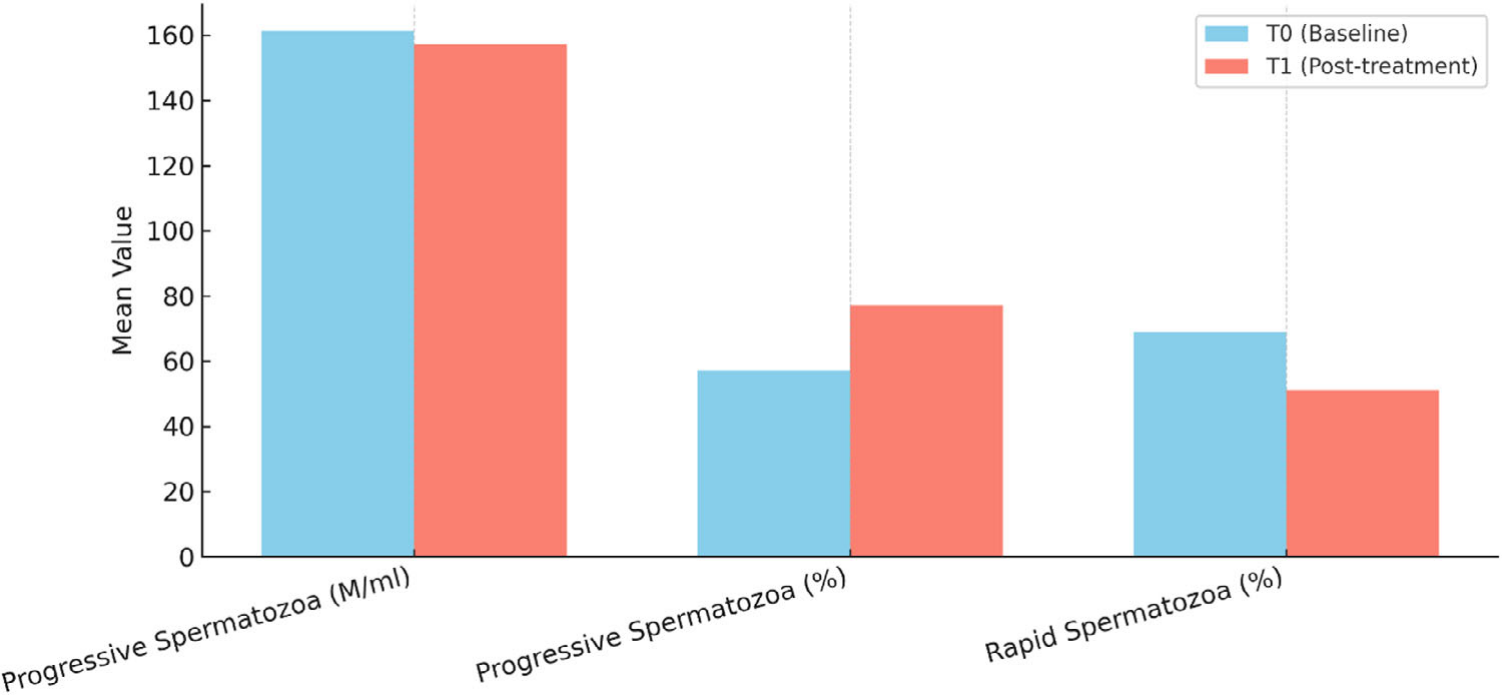
Comparative bar charts of progressive motility (M/mL and %) and percentage of rapid spermatozoa in normospermic group between T0 and T1 (T0 = baseline; T1 = 40 min after pentoxifylline administration).

**FIGURE 5:**
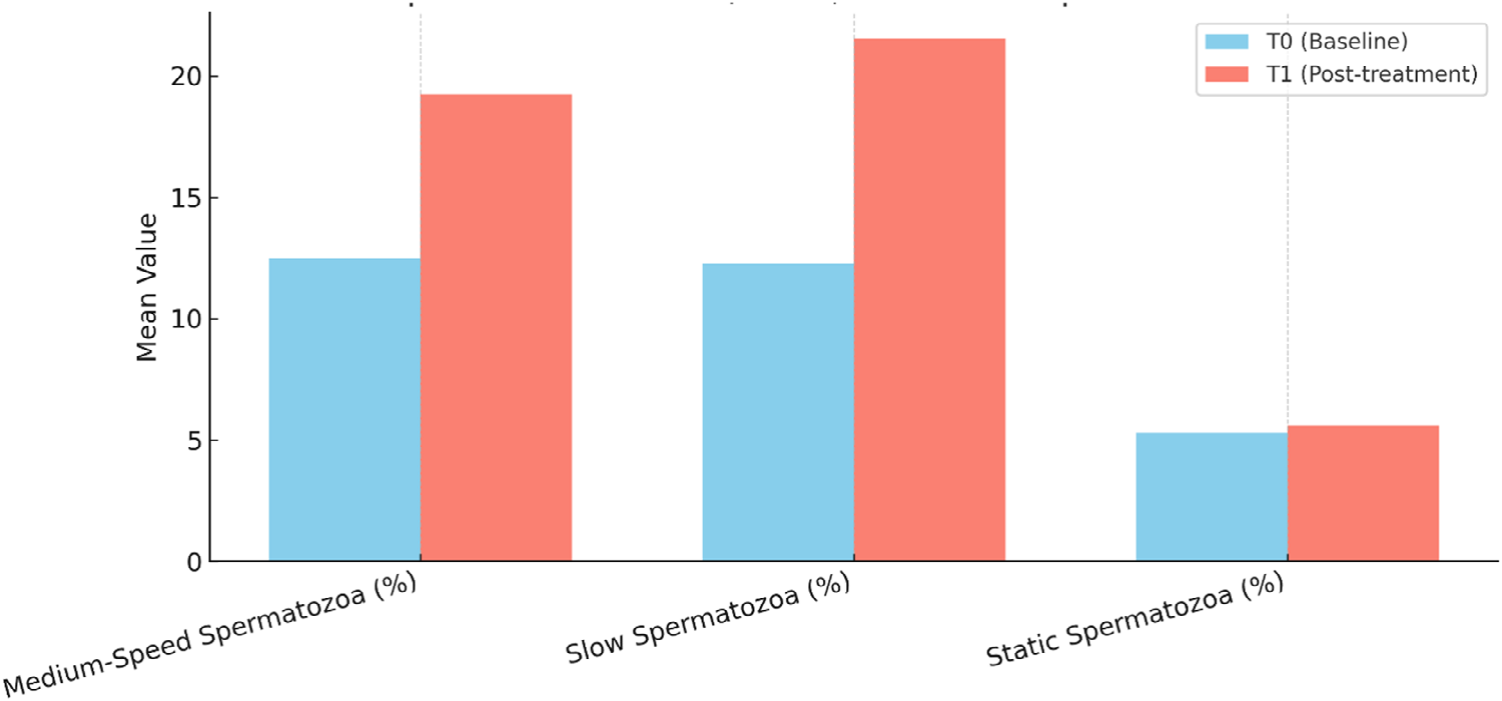
Comparative bar charts of medium‐speed%, slow% and static% spermatozoa in normospermic group between T0 and T1 (T0 = baseline; T1 = 40 min after pentoxifylline administration).

The 29 dogs included in the study were divided into two groups based on sperm concentration at T0: 14 normospermic dogs and 15 oligospermic dogs. No signs of adverse reactions were observed following the administration of pentoxifylline, based on close monitoring of the treated subjects by both owners and veterinarians. Statistical analysis of the subjects revealed improvements across all parameters. Notably, sperm concentration and progressive motility showed statistically significant enhancement. In normospermic dogs, a significant (*p* < 0.05) increase in both sperm concentration and the percentage of motile spermatozoa was observed. The comparison of various parameters at T0 and T1 in normospermic dogs is illustrated in Figures 3, 4, 5.

**FIGURE 6:**
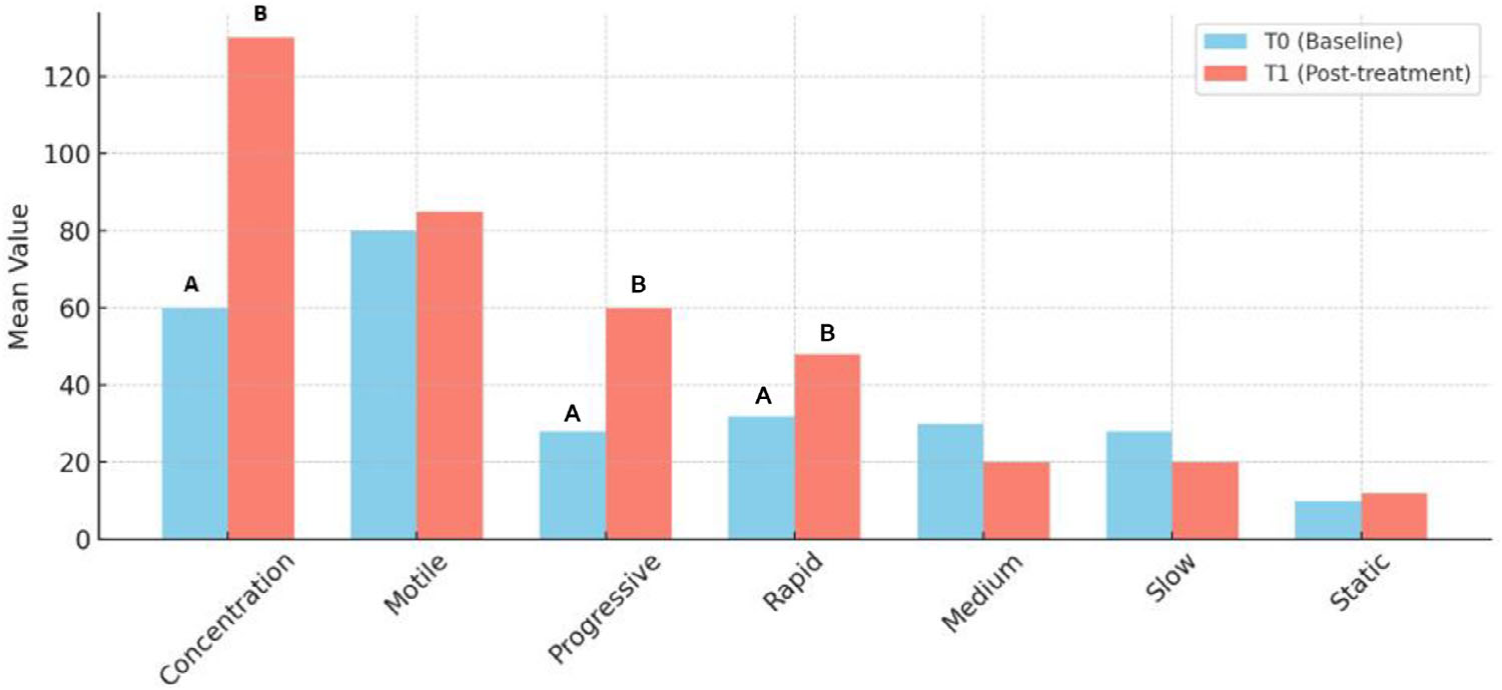
Comparative bar chart of seminal parameters at T0 and T1 (T0 = baseline; T1 = 40 min after pentoxifylline administration) in oligospermic dogs. (A) and (B): *p *< 0.05.

**FIGURE 7:**
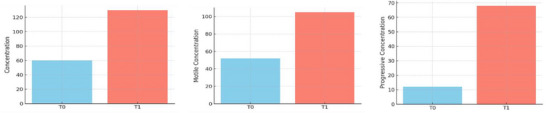
Comparative bar charts of sperm concentration (M/mL), motility concentration (M/mL) and progressive concentration (M/mL) between T0 and T1 (T0 = baseline; T1 = 40 min after pentoxifylline administration) in oligospermic dogs.

In the analysis of oligospermic dogs, a significant increase (*p* < 0.05) was observed in sperm concentration, the percentage of progressively motile sperm, and the proportion of rapid spermatozoa. The comparison of the various parameters at T0 and T1 in oligospermic dogs is illustrated in Figures 6 and 7.

Finally, regarding sperm vitality assessed using the Eosin–Nigrosin staining, the results show a slight increase from T0 to T1 in normospermic subjects, while in oligospermic dogs the average remains unchanged. In both groups, therefore, no statistically significant differences were observed.

**FIGURE 8:**
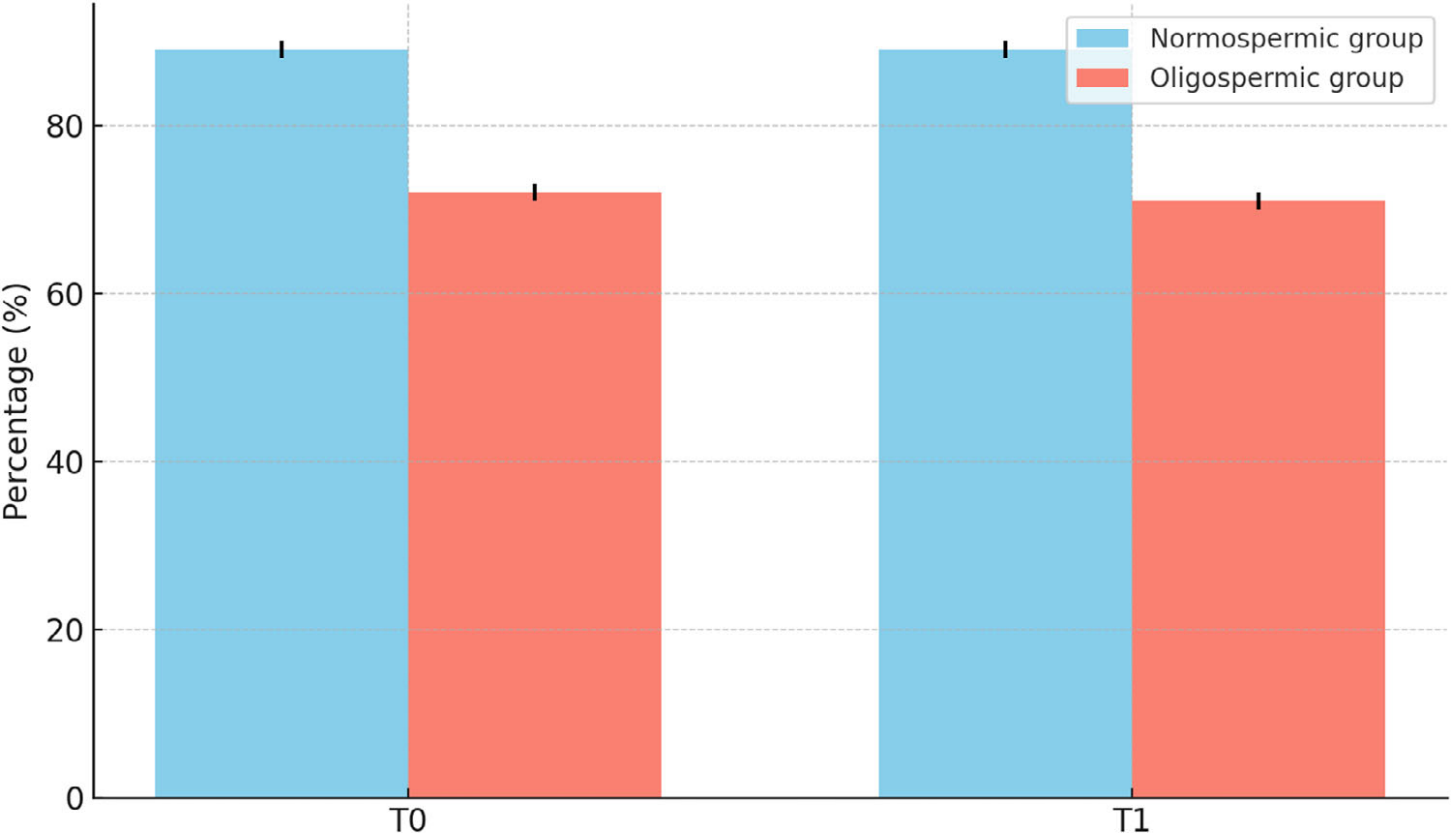
Comparative bar chart of seminal parameters at T0 and T1 (T0 = baseline; T1 = 40 min after pentoxifylline administration) between normospermic and oligospermic groups.

The comparison between T0 and T1 is illustrated in the following Figure 8.

## 4 Discussion

The use of pentoxifylline in the management of male infertility is well‐documented in human medicine, where it is employed both as a long‐term treatment (Pozor et al. 2011) and as a one‐shot administration, due to its rapid gastrointestinal absorption and peak plasma concentration occurring 30–40 min after oral intake (Smith et al. 1986). Numerous studies have demonstrated the role of pentoxifylline in protecting spermatozoa during cryopreservation, enhancing motility and acrosome reaction and reducing oxidative stress by limiting ROS. These effects are associated with improved fertilization rates and conception success (P. Nazari et al. 2020), and have been reported across several species, including rooster (P. Nazari et al. 2020), ram (Hassanpour et al. 2010), dog (Mirshokraei et al. 2011) and stallion (Pozor et al. 2011). In the canine model specifically, pentoxifylline has primarily been studied in vitro as a semen additive, where it has been shown to induce capacitation, trigger acrosome reaction and enhance sperm motility (Mirshokraei et al. 2011). However, no prior studies evaluated the effects of oral administration of pentoxifylline on fresh canine semen.

Pentoxifylline also exerts vasodilatory and anti‐inflammatory effects, which may contribute to improved semen quality. These effects were observed in rams (Fadl et al. 2023), where administration significantly enhanced testicular perfusion and preserved Leydig cell function, essential for testosterone synthesis. Testosterone is crucial for spermatogenesis as it directly stimulates Sertoli cells, which provide metabolic support to developing spermatozoa (Walker 2010).

Another mechanism of pentoxifylline is its antioxidant activity, which limits ROS accumulation and thus safeguards seminiferous tubules—key sites for spermatogenesis. Spermatozoa, being rich in polyunsaturated fatty acids, are particularly susceptible to oxidative damage due to their inherently weak antioxidant defence systems. Excess ROS reduces sperm motility and fusion capacity, induces chemical and structural damage to sperm DNA, and ultimately compromises fertility (Aiudi et al. 2022).

Pentoxifylline also mitigates the ROS‐mediated inactivation of nitric oxide (NO), thereby increasing NO bioavailability, an important factor in sperm capacitation and the acrosome reaction (Pisu et al. 2014; Fadl et al. 2023).

In this study, all dogs showed improved motility, with a significant increase in progressive motility, further supporting pentoxifylline's efficacy in enhancing one of the key predictors of fertilizing potential (Pisu et al. 2014). It's known haemorheological effects, reducing blood and plasma viscosity, enhancing erythrocyte deformability and decreasing fibrinogen levels and platelet aggregation (Cordova et al. 1992; Fadl et al. 2023), may also reduce semen viscosity and facilitate sperm movement. The increase in sperm concentration observed in the present study is unlikely to reflect enhanced spermatogenesis, given the short interval between drug administration and semen collection. Instead, this effect may be attributed to improved sperm release and epididymal emptying, potentially mediated by pentoxifylline's vasodilatory, haemorheological and antioxidant properties. Pentoxifylline‐induced elevation of intracellular cAMP is more plausibly involved in the enhancement of sperm function and motility, through increased mitochondrial activity and energy metabolism, rather than in directly increasing sperm production (Dadgar et al. 2023). Elevated cAMP enhances mitochondrial activity, improves spermatozoal energy metabolism, reduces ROS generation (a major factor in oxidative stress) and improves the ability of spermatozoa to bind to the zona pellucida (P. Nazari et al. 2020).

Importantly, no alterations were observed in sperm viability, as assessed by Eosin–Nigrosin staining, confirming the absence of cytotoxic effects of pentoxifylline on spermatozoa.

In conclusion, the antioxidant, vasodilatory, anti‐inflammatory and cAMP‐elevating actions of pentoxifylline collectively contribute to improved sperm concentration and motility, especially in subfertile individuals. These findings suggest that pentoxifylline may be a useful adjunct therapy not only in managing reduced fertility but also during reproductive periods affected by environmental stressors such as heat (Burgio et al. 2024).

Furthermore, the addition of pentoxifylline to cryopreserved semen has been extensively studied, with post‐thaw improvements (Hassanpour et al. 2010; Guasti et al. 2013; P. Nazari et al. 2020). Thus, pentoxifylline may be effectively used as a protective agent during freeze‐thaw cycles in canine sperm, to mitigate oxidative stress and structural sperm damage.

## 5 Conclusions

This study confirmed that the use of pentoxifylline leads to a significant improvement in semen quality in dogs, particularly regarding sperm concentration and the percentage of progressively motile spermatozoa. The most pronounced improvements were observed in oligospermic subjects compared to normospermic ones. No side effects were detected in the treated dogs, nor were any negative effects observed on semen parameters. The treatment was well tolerated and may represent a promising one‐shot therapeutic option for the clinical management of canine infertility, especially in oligospermic individuals. Indeed, good treatment compliance, absence of adverse effects, ease of administration and rapid onset of action make its application particularly attractive in practical clinical settings. Further studies on a larger sample size, including post‐treatment fertility monitoring and chronic administration, mirroring the protocol used in human male infertility, combined with biomolecular and immunohistochemical analyses, could help confirm the efficacy and clinical applicability of this therapeutic approach in dogs.

## Author Contributions

**Vincenzo Cicirelli**: investigation, methodology, data curation, writing – original draft, writing – review and editing, conceptualization. **Matteo Burgio**: methodology, data curation, writing – review and editing. **Lorenza Frattina**: methodology, writing – review and editing, **Francesco Caprio**: formal analysis, writing – review and editing. **Domenico Robbe**: methodology, writing – review and editing. **Federica Ariu**: methodology, writing – review and editing. **Annalisa Rizzo**: conceptualization, methodology, writing – review and editing, supervision.

## Funding

The authors have nothing to report.

## Ethics Statement

The experimental protocol was reviewed and approved by the Animal Welfare Committee (Protocol No. 10850/2025), in compliance with Directive 2010/63/EU on animal research. All dog owners provided written informed consent prior to enrolment.

## Conflicts of Interest

The authors declare no conflicts of interest.

## Data Availability

The data presented in this study are available on request from the corresponding author.
